# Perinatal testosterone exposure potentiates vascular dysfunction by ERβ suppression in endothelial progenitor cells

**DOI:** 10.1371/journal.pone.0182945

**Published:** 2017-08-15

**Authors:** Weiguo Xie, Mingming Ren, Ling Li, Yin Zhu, Zhigang Chu, Zhigang Zhu, Qiongfang Ruan, Wenting Lou, Haimou Zhang, Zhen Han, Xiaodong Huang, Wei Xiang, Tao Wang, Paul Yao

**Affiliations:** 1 Institute of Burns, Tongren Hospital of Wuhan University, Wuhan, P.R.China; 2 Department of Cardiovascular Surgery, Peking University Shenzhen Hospital, Shenzhen, P.R.China; 3 Department of Pediatrics, Maternal and Child Health Care Hospital of Hainan Province, Haikou, P.R.China; 4 Department of Geriatrics, National Key Clinical Specialty, Guangzhou First People's Hospital, Guangzhou Medical University, Guangzhou, P.R.China; 5 School of Life Sciences, Hubei University, Wuhan, P.R.China; Centro Cardiologico Monzino, ITALY

## Abstract

Recent clinical cohort study shows that testosterone therapy increases cardiovascular diseases in men with low testosterone levels, excessive circulating androgen levels may play a detrimental role in the vascular system, while the potential mechanism and effect of testosterone exposure on the vascular function in offspring is still unknown. Our preliminary results showed that perinatal testosterone exposure in mice induces estrogen receptor β (ERβ) suppression in endothelial progenitor cells (EPCs) in offspring but not mothers, while estradiol (E2) had no effect. Further investigation showed that ERβ suppression is due to perinatal testosterone exposure-induced epigenetic changes with altered DNA methylation on the ERβ promoter. During aging, EPCs with ERβ suppression mobilize to the vascular wall, differentiate into ERβ-suppressed mouse endothelial cells (MECs) with downregulated expression of SOD2 (mitochondrial superoxide dismutase) and ERRα (estrogen-related receptor α). This results in reactive oxygen species (ROS) generation and DNA damage, and the dysfunction of mitochondria and fatty acid metabolism, subsequently potentiating vascular dysfunction. Bone marrow transplantation of EPCs that overexpressed with either ERβ or a SIRT1 single mutant SIRT1-C152(D) that could modulate SIRT1 phosphorylation significantly ameliorated vascular dysfunction, while ERβ knockdown worsened the problem. We conclude that perinatal testosterone exposure potentiates vascular dysfunction through ERβ suppression in EPCs.

## Introduction

Testosterone (T) is the principal male sex hormone. Recent retrospective national cohort study shows the association of testosterone therapy with mortality, myocardial infarction, and stroke in men with low testosterone levels [1], indicating that excessive testosterone exposure may be detrimental to the cardiovascular system [2–4]. The polycystic ovary syndrome (PCOS) in women displays high circulating androgen levels, and demonstrates structural and functional abnormalities in cardiovascular system [5]. It has been reported that excessive circulating androgen levels may affect the fetus and increase the risk of mood disorders in offspring [6], while the potential mechanism and effect of perinatal testosterone exposure on vascular dysfunction in offspring is still unknown.

Endothelial progenitor cells (EPCs) support the integrity of the vascular endothelium, and the number and function of EPCs correlate inversely with cardiovascular risk factors [7, 8]. It has been reported that EPCs mobilize to the vascular wall and differentiate into endothelial cells during aging and vascular injury, while the detailed mechanism still needs to be fully understood [9, 10].

Estrogen receptor β (ERβ) is the principal estrogen receptor expressed in the vascular system. It is responsible for the basal expression of SOD2 [11] and ERRα [12], and contributes to vascular function [13]. ERβ suppression downregulates the expression of SOD2 (mitochondrial superoxide dismutase) and ERRα (estrogen-related receptor α), where decreased SOD2 expression results in reactive oxygen species (ROS) generation and DNA damage, and decreased ERRα expression results in dysfunction of mitochondria and fatty acid metabolism, which subsequently potentiates vascular dysfunction. We have previously reported that SIRT1-mediated ERβ suppression in the endothelium contributes to vascular dysfunction, and the modulation of SIRT1 phosphorylation through a single mutant SIRT1-C152(D) restores ERβ suppression and ameliorates this effect [14].

Our preliminary data showed that perinatal testosterone exposure induces significant ERβ suppression in EPCs in offspring, while E2 exposure has no effect. Further investigation showed that ERβ suppression is due to testosterone-induced epigenetic changes with altered DNA methylation on the ERβ promoter, and this kind of suppression lasts for a long time until old age. During aging, the ERβ-suppressed EPCs mobilize to the vascular wall and differentiate into endothelial cells, which then contributes to vascular dysfunction. To further prove this, the bone marrow derived mononuclear cells (MNCs) were infected by Tie2-driven lentivirus that is specific for modulation of ERβ expression in EPCs, and then they were used for bone marrow transplantation (BMT). We found that overexpression of either ERβ or a SIRT1 single mutant SIRT1-C152(D), significantly ameliorated vascular dysfunction, while ERβ knockdown worsened the problem. We conclude that perinatal testosterone exposure potentiates vascular dysfunction through ERβ suppression in EPCs.

## Materials and methods

### Materials

The antibodies for ERRα (ab37438), H2AX (ab20669), γH2AX (ab2893), H3K9me2 (ab1220), H3K9me3 (ab8898) and H3K27me3 (ab6002) were obtained from Abcam, and all the other antibodies, including β-actin (sc-47778), eNOS (sc-654), ERβ (sc-137381) and SOD2 (sc-30080) were obtained from Santa Cruz Biotechnology. The mitochondrial fraction was isolated using a Pierce Mitochondria Isolation Kit (Pierce Biotechnology) according to manufacturers’ instructions. Nuclear extracts were prepared using the NE-PER Nuclear and Cytoplasmic Extraction Reagents Kit (Pierce Biotechnology). Protein concentration was measured using the Coomassie Protein Assay Kit (Pierce Biotechnology) according to manufacturers’ instructions.

### Generation of lentivirus

#### Tie2-driven ERβ/SIRT1-C152(D) expression lentivirus

The mouse genomic DNA was purified from C57BL/J6 wild type mouse, and the endothelium-specific Tie2 promoter (-1.2kb upstream) was amplified by PCR. The mouse cDNA for ERβ and SIRT1 was obtained from Open Biosystems. The cDNA for SIRT1 single mutant SIRT1-C152(D) was made from SIRT1 wild type (SIRT1-WT) using the Site-directed Mutagenesis Kit (Promega), and the SIRT1 amino acid at 152 was mutated from C (coded by TGT) to D (coded by GAT). The Tie2 promoter was fused with the mouse ERβ, or SIRT1-C152(D) cDNA, and then subcloned into the pLVX-Puro vector (from Clontech) with the restriction sites of Xho1 and Xba1 using the below primers: ERβ forward primer: 5’-gtac-ctcgag-cag att gga agc att aca ggc-3’ (Xho1) and ERβ reverse primer: 5’-gtac-tctaga-agg ccc acg atg cta ggg tac-3’ (Xba1). SIRT1 forward primer: 5’-gtac-ctcgag- atg gcg gac gag gtg gcg ctc -3’ (Xho1) and SIRT1 reverse primer: 5’-gtac-tctaga- tta tga ttt gtc tga tgg ata -3’ (Xba1). The Tie2-empty, Tie2-ERβ or Tie2-SIRT1-C152(D), and the parallel positive control pLVX-AcGFP1-C1 (#632155, Clontech) were expressed through Lenti-X^™^ Lentiviral Expression Systems (from Clontech) according to manufacturers’ instructions. The harvested virus was concentrated and diluted to get a titer of 1×10^7^ cfu/ml. In order to evaluate the efficiency of SIRT1 lentivirus infection in the EPCs or MECs on the vascular wall, the primers specific for SIRT1-WT and SIRT1-C152(D) were designed as follows to distinguish the expression differences by qPCR. SIRT1-WT forward: 5’-gct ttc att cc TGT gaa agt ga -3’, SIRT1-WT reverse: 5’-ttt aag aat tgt tcg agg atc g-3’; SIRT1-C152(D) forward: 5’-gct ttc att cc GAT gaa agt ga -3’, SIRT1-C152(D) reverse: 5’-ttt aag aat tgt tcg agg atc g-3’ [14].

#### Tie2-driven ERβ shRNA lentivirus

According to our preliminary data from *in vitro* cell culture experiments, the following sequence was confirmed as the most effective to knockdown mouse ERβ: 5’-ccg gag aac ggt gtg gtc atc aaa tct cga gat ttg atg acc aca ccg ttc ttt ttt tg-3’, and the shRNA template for ERβ or scrambled were designed (sense strand + loop + antisense strand) and the related double strand DNA (dsDNA) was synthesized and annealed. They were fused with mouse Tie2 promoter (-1.2kb upstream) by BamH1 sticky site at 5’-end and EcoR1 sticky site at 3’-end, and then inserted into pLVX-shRNA1 vector (from Clontech) using BamH1/EcoR1 restriction sites. The Tie2-scrambled (EMP) or Tie2-shERβ lentivirus was then expressed through Lenti-X^™^ shRNA Expression Systems (from Clontech) according to manufacturers’ instructions.

### In vivo mouse experiments

The animal protocol conformed to US NIH guidelines (Guide for the Care and Use of Laboratory Animals, No. 85–23, revised 1996), and was reviewed and approved by the Institutional Animal Care and Use Committee (from Wuhan University). The C57Bl/6 mice were housed 4 or 5 per cage on a 12:12-h light-dark cycle and were given phytoestrogen-free commercial rodent chow and water ad libitum on arrival.

**Animal Protocol 1**: The 2-month female mice were anaesthetized by intraperitoneal injection of 100mg/kg ketamine/16mg/kg xylazine, and received treatments consisting of 60-day time release pellets (Innovative Research of America) that were implanted subcutaneously via a ~3mm incision on the dorsal aspect of the neck. Hormone pellets contained 5mg of either dihydrotestosterone (DHT, #A-161), estradiol (E2, #E-121), combined DHT and E2 (DHT/E2), or vehicle pellets (CTL) containing the same matrix but with no hormone [15]. After 1 week of surgery recovery, the female mice mated with male mice, and successful pregnancy was confirmed by examining the vaginal plugs. The pregnant mice were kept fed until baby delivery (~3 weeks) with another 3-week of lactation for the offspring. The mothers were sacrificed, and the blood was collected for analysis of hormones by radioimmunoassay. The EPCs were isolated from both bone marrow and peripheral blood [16] for gene expression analysis. The male offspring were selected and fed with high-fat diet (HFD, 60% calories from fat, Research Diets, #D12492) throughout the experiments. Parts of the young (4 months) and old (20 months) offspring were overnight-fasted, euthanized by 100mg/kg pentobarbital, and the EPCs were isolated for gene expression analysis. The EPCs numbers were counted [17] and the EPCs characteristics for colony form unit (CFU) [18] and migration [16] were evaluated. The tissues, including liver, heart and aorta were isolated for gene expression analysis or in vivo lipid uptake, and the blood was collected for measuring lipids, including total cholesterol, triglyceride, LDL and HDL cholesterol [14]. The MECs from the aorta were isolated for in vitro cell culture analysis [19, 20], and the MECs from the thoracic aortas [21] were picked up by Laser Capture Microdissection (LCM) for mRNA analysis, and the vascular function was evaluated by vessel tension [22, 23] and blood pressure [24–26] as described previously in our lab [14].

**Animal Protocol 2**: The male offspring from CTL or DHT group in Animal Protocol 1 were used as recipients for bone marrow transplantation (BMT). Bone marrow cells were harvested from the tibias and femurs of the male offspring (4 months old) that were obtained from CTL group in Animal Protocol 1 as the donor for BMT. The isolated MNCs were purified by density centrifugation using Histopaque 1083^®^ (#-1083-1, Sigma), and resuspended in 10ml of RPMI 1640 supplemented with 10% FBS and 2mM EDTA. We then added 4μg/ml of final concentration of polybrene with 100μl of concentrated lentivirus (1×10^7^ cfu/ml), which included either Tie2-EMP, or Tie2-ERβ, or Tie2-SIRT1-C152(D) or Tie2-shERβ. We incubated the cells for 6 hours to achieve maximum 100% viral infection of cells. A parallel viral infection on the same MNCs cells was achieved using 100μl of 1×10^7^ cfu/ml of pLVX-AcGFP1-C1 virus (#632155, Clontech) that can express GFP (green fluorescent proteins). The efficiency of viral infection was confirmed as ~100% using fluorescent microscopy. The above lentivirus infected MNCs were washed twice and resuspended by PBS, and then systemically transplanted (2×10^6^ cells) into the above recipient male offspring (with CTL or DHT group) that had been lethally irradiated with 2 doses of 6 Gy 3 hours apart. All transplant-recipient mice were set aside for a minimum of 4 weeks to allow for complete reconstitution of the bone marrow [10], see details in S1 Table. The bone marrow transplanted mice were overnight-fasted and euthanized at the age of 20 months. The EPCs and MECs were isolated for in vitro cell culture analysis, or the MECs and SMCs (smooth muscle cells) were isolated by LCM for mRNA analysis, and parts of the mice were used for vascular function analysis.

### Hormone measurement by radioimmunoassay (RIA)

The blood samples were collected and the plasma was prepared and kept frozen until steroids were measured with RIA kits. A 25μl aliquot of each sample was diluted in 475μl of 0.1M PBS and sent to the Analysis Core Facility in our institute (Wuhan University) for RIA using commercially available kits (from Beckman Coulter) for estradiol (#DSL-39100) and total testosterone (TT, #DPC-TKTT2).

### Isolation of endothelial progenitor cells (EPCs)

For the isolation of circulating EPCs, the mononuclear cells were isolated from 500μl of peripheral blood [16], while for bone marrow- derived EPCs, the primary bone marrow cells were collected from tibia in treated mice. The isolated cells were maintained in endothelial basal medium (EBM-2) with supplements of hydrocortisone, EGF, and 10% FCS on fibronectin/gelatin-coated dishes. The media were refreshed every day for 3 days, and then the cells were stimulated with human recombinant VEGF for 2 days as described previously [17]. The nonadherent cells were removed and collected every day as the non-EPCs cells. The adherent cells were characterized by washing with medium and incubating with 2.4ug/ml 1,1′-dioctadecyl-3,3,3′,3′-tetramethylindocarbocyanine-labeled acetylated LDL (Dil-Ac-LDL) for 1 hour. Cells were fixed in 2% paraformaldehyde and counterstained with FITC-labeled lectin. Double positive staining cells were considered to be endothelial progenitor cells (EPCs), and the numbers of circulating EPCs were counted in 10 randomly selected high-power fields (HPF) under a fluorescent microscope [17]. After 4 days in culture, nonadherent cells were removed, and the adherent cells were reseeded at a density of 5×10^4^/cm2. After 3 days in further culture, the cells were used as EPC-rich cell population for western blot analysis.

### EPCs colony forming unit (EPCs-CFU) assay

The mononuclear cells (MNCs) isolated from peripheral blood were resuspended in EBM-2 medium with all the supplements and plated on fibronectin-coated 6-well plates at a concentration of 5×10^6^ cells per well. This step was designed to remove mature circulating endothelial cells, which rapidly adhere to fibronectin. After 2-day culturing, the nonadherent cells were aspirated and counted, and then reseeded on fibronectin-coated 24-well plates at a concentration of 1×10^6^ cells per well. The medium was changed on day 5 of assay, and on day 7 the endothelial colonies were counted manually. Strict guidelines were followed to ensure consistent counting of EPC colonies. Colonies were only counted as EPCs-CFU if they consisted of more than 50 cells and contained a core of rounded cells with flat, spindle-shaped cells emanating from the periphery [18].

### EPCs migration assay

EPCs migration was evaluated using a modified Boyden chamber as previously described [16]. The EPCs (2.5×10^5^) were plated in 500 μL medium in each well. The EBM-2 medium supplemented with 20% FBS was added to the upper chamber of a transwell plate and EBM-2 medium supplemented with 20% FBS and 10ng/mL VEGF was added to the lower chamber. After incubation for 6 hours, cells that had migrated to the lower surface of the membrane were fixed with 2% paraformaldehyde and stained with DAPI for 15 min at room temperature and then washed twice with PBS, and the cells were counted manually.

### Isolation of mouse endothelial cells (MECs)

Isolation of endothelial cells from the aorta was performed following the previously described procedure [19, 27]. The isolated MECs were further characterized by immunofluorescence staining with an antibody to the von Willebrandt factor (vWF), and the P3 to P5 passages were used. Isolated MECs were maintained in DME medium with all of the endothelial cell supplements, plus charcoal-stripped Fetal Bovine Serum (#12676029, Life Technologies) to remove traces of interfering basal estrogen.

### RT reaction and real-time quantitative PCR

Total RNA from treated cells was extracted using the RNeasy Mini Kit (for small amounts of cells isolated using laser capture microdissection techniques) or the RNeasy Micro Kit (Qiagen), and the RNA was reverse transcribed using an Omniscript RT kit (Qiagen). All of the primers were designed using Primer 3 Plus software with the Tm as 60°C, primer size as 21bp, and the product length in the range of 140-160bp (see S2 Table). The primers were validated with the amplification efficiency in the range of 1.9–2.1, and the amplified products were confirmed with agarose gel. The real-time quantitative PCR was run on iCycler iQ (Bio-Rad) with the Quantitect SYBR green PCR kit (Qiagen). The PCR was performed by denaturing at 95°C for 8 min, followed by 45 cycles of denaturation at 95°C, annealing at 60°C, and extension at 72°C for 10s, respectively. 1 μl of each cDNA was used to measure target genes. The β-actin was used as the housekeeping gene for transcript normalization, and the mean values were used to calculate relative transcript levels with the ^ΔΔ^CT method according to instructions from Qiagen. In brief, the amplified transcripts were quantified by the comparative threshold cycle method using β-actin as a normalizer. Fold changes in gene mRNA expression were calculated as 2^−ΔΔCT^ with CT = threshold cycle, ΔCT = CT(target gene)-CT(β-actin), and the ΔΔCT = ΔCT(experimental)-ΔCT (reference).

### Western blotting

Cells were lysed in an ice-cold lysis buffer (0.137M NaCl, 2mM EDTA, 10% glycerol, 1% NP-40, 20mM Tris base, pH 8.0) with protease inhibitor cocktail (Sigma). The proteins were separated in 10% SDS-PAGE and further transferred to the PVDF membrane. The membrane was incubated with appropriate antibodies, washed and incubated with HRP-labeled secondary antibodies, and then the blots were visualized using the ECL+plus Western Blotting Detection System (Amersham). The blots were quantitated by IMAGEQUANT, and final results were normalized by β-actin.

### Chromatin Immunoprecipitation (ChIP)

Cells were washed and crosslinked using 1% formaldehyde for 20 min and terminated by 0.1M glycine. Cell lysates were sonicated and centrifuged. 500μg of protein were pre-cleared by BSA/salmon sperm DNA with preimmune IgG and a slurry of Protein A Agarose beads. Immunoprecipitations were performed with the indicated antibodies, BSA/salmon sperm DNA and a 50% slurry of Protein A agarose beads. Input and immunoprecipitates were washed and eluted, then incubated with 0.2mg/ml Proteinase K for 2h at 42°C, followed by 6h at 65°C to reverse the formaldehyde crosslinking. DNA fragments were recovered by phenol/chloroform extraction and ethanol precipitation. A 151bp fragment in the range of -400~-200 from the transcription start site on mouse ERβ promoter was amplified by real-time PCR (qPCR) using the below primers: forward 5’- tcg gtg cta tta ccc gaa ac-3’ and reverse 5’- cca ggg att ctg gac tta acc-3’.

### DNA methylation analysis

We developed a real-time PCR based method for methylation specific PCR (MSP) analysis on mouse ERβ promoter according to the previously described method with some modifications [28–30]. The mouse genomic DNA from EPCs was extracted and purified, then treated by bisulfite modification using EpiJET Bisulfite Conversion Kit (#K1461, Fisher). The modified DNA was then amplified using methylated and unmethylated primers for MSP that were designed using the Methprimer software: (http://www.urogene.org/cgi-bin/methprimer/methprimer.cgi) with the below details: Methylated primer Forward 5’-TGG TTT TTT TGA AAG GTA TTT TCG-3’, Reverse 5’-TAC CAA TAA CCG CAC AAA CCG-3’; Unmethylated primer Forward 5’- TGG TTT TTT TGA AAG GTA TTT TTG-3’; Reverse 5’-CCA ATA ACC ACA CAA ACC ACT-3’. The product size: 131bp (methylated) & 133bp (unmethylated); CpG island size: 124bp; Tm: 57–63°C; GC%>54. The final methylation readout was normalized by unmethylated input PCR, the PCR products were also confirmed by electrophorese using 2% agarose gel and the DNA bands were imaged.

### Measurement of ROS generation and DNA damage

Treated cells were seeded in a 96-well plate and incubated with 10μM CM-H2DCFDA (Invitrogen) for 45min at 37°C. Then, the intracellular formation of reactive oxygen species (ROS) was measured at excitation/emission wavelengths of 485/530nm using a FLx800 microplate fluorescence reader (Bio-Tek), and the data was normalized as arbitrary units [31]. 3-nitrotyrosine (3-NT) was measured by 3-Nitrotyrosine ELISA Kit (ab116691 from abcam) according to manufacturers’ instructions. The DNA damage was evaluated through formation of γH2AX from nuclear extracts by western blotting using H2AX as input control.

### Evaluation of mitochondrial function

#### Mitochondrial DNA copies

The genomic DNA was extracted from treated MECs using a QIAamp DNA Mini Kit (Qiagen) and the mitochondrial DNA was extracted using the REPLI-g Mitochondrial DNA Kit (Qiagen). The purified DNA was used for the analysis of genomic β-actin (marker of the nuclear gene) and ATP6 (ATP synthase F0 subunit 6, marker of the mitochondrial gene) respectively using the qPCR method as mentioned above. The primers for genomic β-actin: forward 5’-acc aca gct gag agg gaa atc-3’ and reverse: 5’-cgt tgc caa tag tga tga cct -3’. The primers for ATP6: forward 5’-cgt aat tac agg ctt ccg aca-3’ and reverse 5’-ctg taa gcc gga ctg cta atg -3’. The mitochondrial DNA copies were obtained from relative ATP6 copies that were normalized by β-actin copies using the ^ΔΔ^CT method.

#### Intracellular ATP level

The intracellular ATP level was determined using the luciferin/ luciferase-induced bioluminescence system. An ATP standard curve was generated at concentrations of 10^−12^–10^−3^M. Intracellular ATP levels were calculated and expressed as nmol/mg protein [31].

#### The OXPHOS proteins

The OXPHOS proteins were measured using theTotal OXPHOS Rodent WB Antibody Cocktail (#ab110413, from Abcam) according to manufacturers’ instructions.

### Evaluation of fatty acid metabolism

#### In vitro lipid transport assay

Cells were seeded in a 12-well plate and grew until they were 80% confluent. After treatment, 0.5mCi well^-1^ of ^14^C-oleic acid (OA) from PerkinElmer was added. After 4h of incubation, the cells were washed and harvested, and the total radioactivity was quantitated by scintillation counting [32].

#### Rate of fatty acid oxidation

The fatty acid oxidation (FAO) rate was measured by evaluation of palmitate oxidation according to the published methods with minor modifications [33, 34]. In brief, the MECs from the heart and aorta in treated mice were cultured in a T25 flask until they were 80% confluent, and the cells were starved for 2h in DMEM medium. Then, they were incubated in DMEM containing 0.5% BSA/0.2mM palmitate/0.5μCi/mL 1-^14^C-palmitate at 37°C for 2h. The flasks were sealed at the beginning of the incubation with a stopper containing a filter (Whatman GF/B paper) pre-soaked in 5% NaOH. The incubation was stopped by the injection of 0.2 ml of 40% perchloric acid into each flask via a needle through the cap to acidify the medium and liberate the CO_2_. After overnight isotopic equilibration at room temperature, filters were removed, and the trapped ^14^CO_2_ and ^14^C acid-soluble products generated by the oxidation of [^14^C]palmitate were counted to calculate total palmitate oxidation. The protein concentrations were measured and the results were expressed as nmol per mg proteins per hour (nmol/mg/h).

#### In vivo fatty acid uptake

Mice were given a bolus dose of 2mCi of ^14^C-OA dissolved in olive oil through oral gavage. One hour after gavage, blood samples were drawn from the tail vein for plasma analysis of radioactivity. Two hours after gavage, the mice were anaesthetized and perfused with PBS, and the heart, aorta and liver were dissected and dissolved overnight at 50μC in tissue solubilizer (1ml per 100mg tissue), then neutralized with 30μlml^-1^ glacial acetic acid. The total radioactivity was quantitated by scintillation counting [32].

#### Plasma analysis for lipids

The total cholesterol (TC), triglyceride (TG), LDL and HDL cholesterol in plasma was measured using a GM7 Micro-Stat Rapid Multiassay Analyser (Analox) according to manufacturers’ instructions.

### Monitoring of the vascular function

Vessel tension for the carotid artery in mice was measured in the Multi Wire Myograph System Model 620M (from Danish Myo Technology, Denmark). The three 3-mm aortic rings from each animal were quickly excised and placed in a Krebs bicarbonate buffer (118 mmol/L NaCl, 4.7mmol/L KCl, 25 mmol/L NaHCO3,1.2 mmol/L KH2PO4, 1.2 mmol/L MgSO4, 2.5 mmol/L CaCl2, and 5 mmol/L glucose), and the adhering tissue and fat were removed. Each ring was positioned between two 40-μm stainless steel wires in an 8-ml organ myograph chamber (DMT 620M), filled with Krebs bicarbonate buffer, maintained at 37±0.5°C and aerated with 95% O_2_ plus 5% CO_2_ (pH = 7.4). At the beginning of the experiment, each vessel ring was stretched to its optimal resting tension and allowed to equilibrate for 1hr. To study vasodilator responses, the acetylcholine (Ach,10^−10^–10^−4^mol/l) induced vasodilation was assessed in aortas preconstricted with phenylephrine (10^−5^mol/l) at a level corresponding at least to the maximal response to potassium (100mmol/l KCl). The dose-response relaxation was measured for cumulative increments of acetylcholine at 1min intervals, and the Ach-induced change in tension was expressed as the percentage of the initial contraction induced by phenylephrine [22, 23].

### Radiotelemetric blood pressure monitoring in *vivo*

Blood pressure was measured in conscious mice with the radiotelemetry technique described previously. In brief, the mice were anaesthetized by intraperitoneal injection of 100mg/kg ketamine/16mg/kg xylazine, a catheter (PE10 tubing) and the telemetry transmitter unit (TA11PA-C10, Data Sciences International (DSI)) was implanted in the left carotid artery, while the radiotransmitter was placed in a subcutaneous pouch along the flank. Mice were treated with analgesics for 3 days (buprenorphine, 0.1 mg/kg) to relieve the pain and were allowed to recover for 7 days after surgery to regain their normal circadian rhythms before blood pressure measurements. While the blood pressure was being monitored, the mice were housed in a quiet room in individual cages placed above the telemetric receivers with an output to a computer. Blood pressure was measured for 5 minutes every hour, processed, and analyzed using the DataQuest ART system (DSI) [24–26].

### Statistical analysis

The data was given as mean ± SEM, and all the experiments were performed at least in quadruplicate unless otherwise indicated. The power calculation was conducted to determine the sample size. The one-way ANOVA and the Turkey—Kramer test was used to determine statistical significance of different groups by SPSS 18 software, and a *P* value < 0.05 was considered significant.

## Results

### Perinatal testosterone exposure suppresses ERβ expression and its target genes in EPCs in young offspring (2 months old), but has no effect on mothers

**Fig 1:**
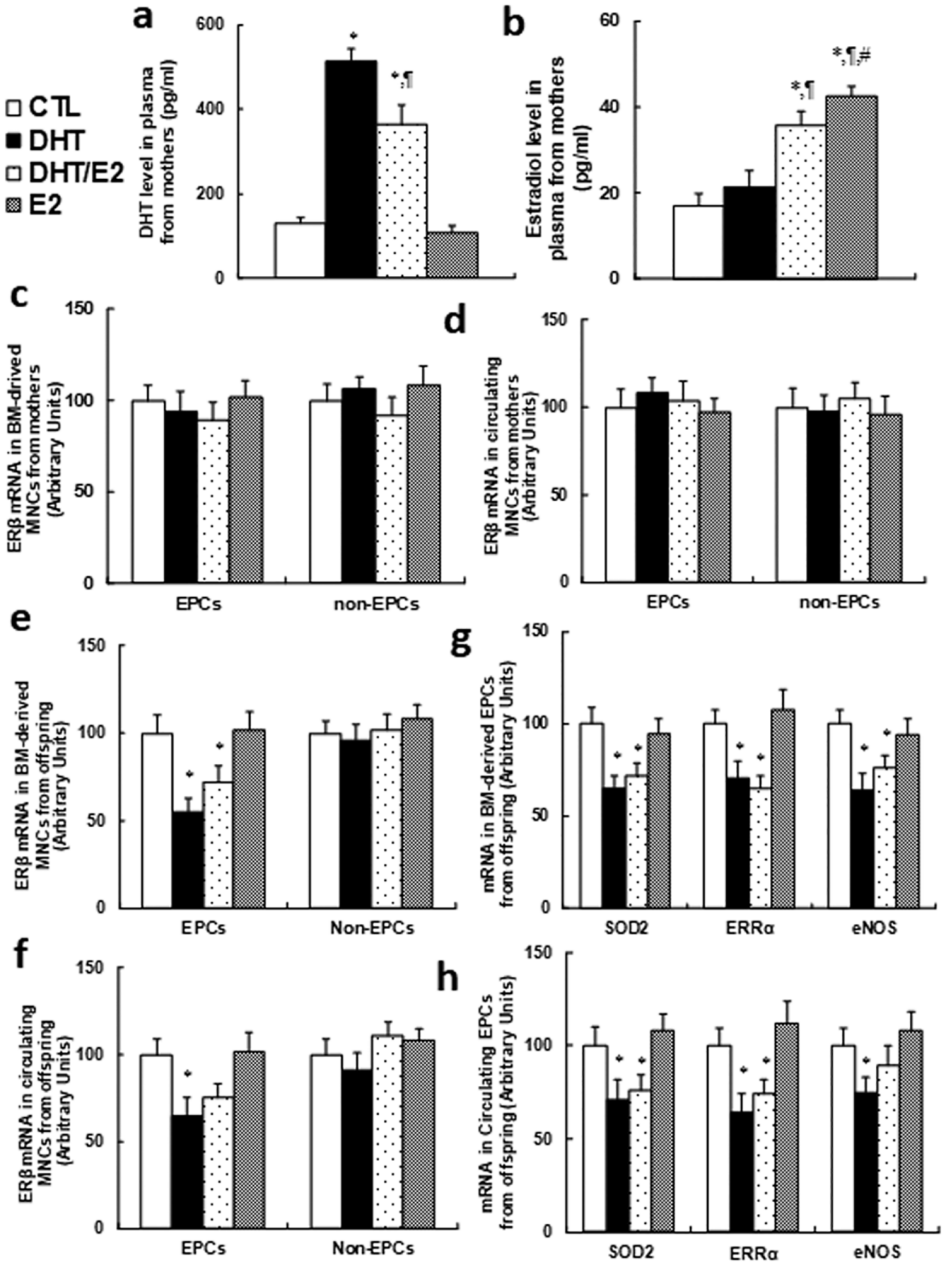
Perinatal testosterone exposure suppresses ERβ expression and its target genes in EPCs in young offspring (2 months old). 2-month old female mice were exposed to 5mg of 60-day release hormone pellets that contained either dihydrotestosterone (DHT) alone, estradiol (E2) alone, combined DHT and E2 (DHT/E2), or controlled vehicle (CTL) during a 7-week perinatal period. The mothers were sacrificed to measure the plasma hormone levels, and the MNCs (including EPCs and non-EPCs) were isolated from either the bone marrow or peripheral blood for analysis of gene expression. The male offspring was also sacrificed at 2 months old for isolation of MNCs (including EPCs and non-EPCs) for further analysis. (a) Dihydrotestosterone (DHT) level in plasma from mothers, n = 8. (b) The estradiol (E2) level in plasma from mothers, n = 8. (c) The ERβ mRNA in BM-derived MNCs from mothers, n = 7. (d) The ERβ mRNA in Circulating MNCs from mothers, n = 7. (e)The ERβ mRNA in BM-derived MNCs from male offspring, n = 6. (f) The ERβ mRNA in Circulating MNCs from male offspring, n = 6. (g) The mRNA levels in BM-derived EPCs from male offspring, n = 7. (h) The mRNA levels in Circulating EPCs from male offspring, n = 7. *, *P*<0.05, vs CTL group; ¶, *P*<0.05, vs DHT group; #, *P*<0.05, vs DHT/E2 group. Results are expressed as mean ± SEM.

We first measured the sex hormone levels in plasma from mothers. In Fig 1a, the dihydrotestosterone (DHT) level was significantly increased in DHT treatment alone or combined DHT/E2 group, while E2 alone showed no effect, reflecting a successful DHT hormone treatment. In Fig 1b, the estradiol level was increased significantly in the DHT/E2 and E2 group compared to CTL group, indicating a successful E2 treatment. We then measured the effect of hormone treatment on ERβ expression in MNCs from mothers, and the results showed that both bone marrow(BM)-derived (see Fig 1c) and circulating MNCs (see Fig 1d) had no difference in both EPCs and non-EPCs. On the other hand, both DHT and T/E2 treatment significantly suppressed ERβ expression in both BM-derived (see Fig 1e) and circulating EPCs (see Fig 1f) in young male offspring (2 months old), while ERβ expression had no effect on non-EPCs. We also measured the ERβ target genes, including SOD2, ERRα and eNOS, and found that their expression was suppressed on both BM-derived (see Fig 1g) and circulating EPCs (see Fig 1h) in both DHT and DHT/E2 treatment, while E2 treatment showed no effect. This suggests that perinatal testosterone exposure has no effect on mothers, while it can significantly suppress ERβ expression in EPCs from young offspring, and E2 seems have no effect. Finally, we evaluated the mobilization characteristics of circulating EPCs from young offspring. The results showed that perinatal exposure of hormones had no effect on circulating EPCs numbers (see Panel A in S1 Fig), EPCs colony forming unit (CFU) (see Panel B in S1 Fig) or EPCs migration (see Panel C in S1 Fig) in young offspring, indicating that perinatal exposure of hormones does not directly affect EPCs mobilization.

### Perinatal testosterone exposure induces ERβ suppression in EPCs through increased methylation on the ERβ promoter, while E2 has no effect

**Fig 2:**
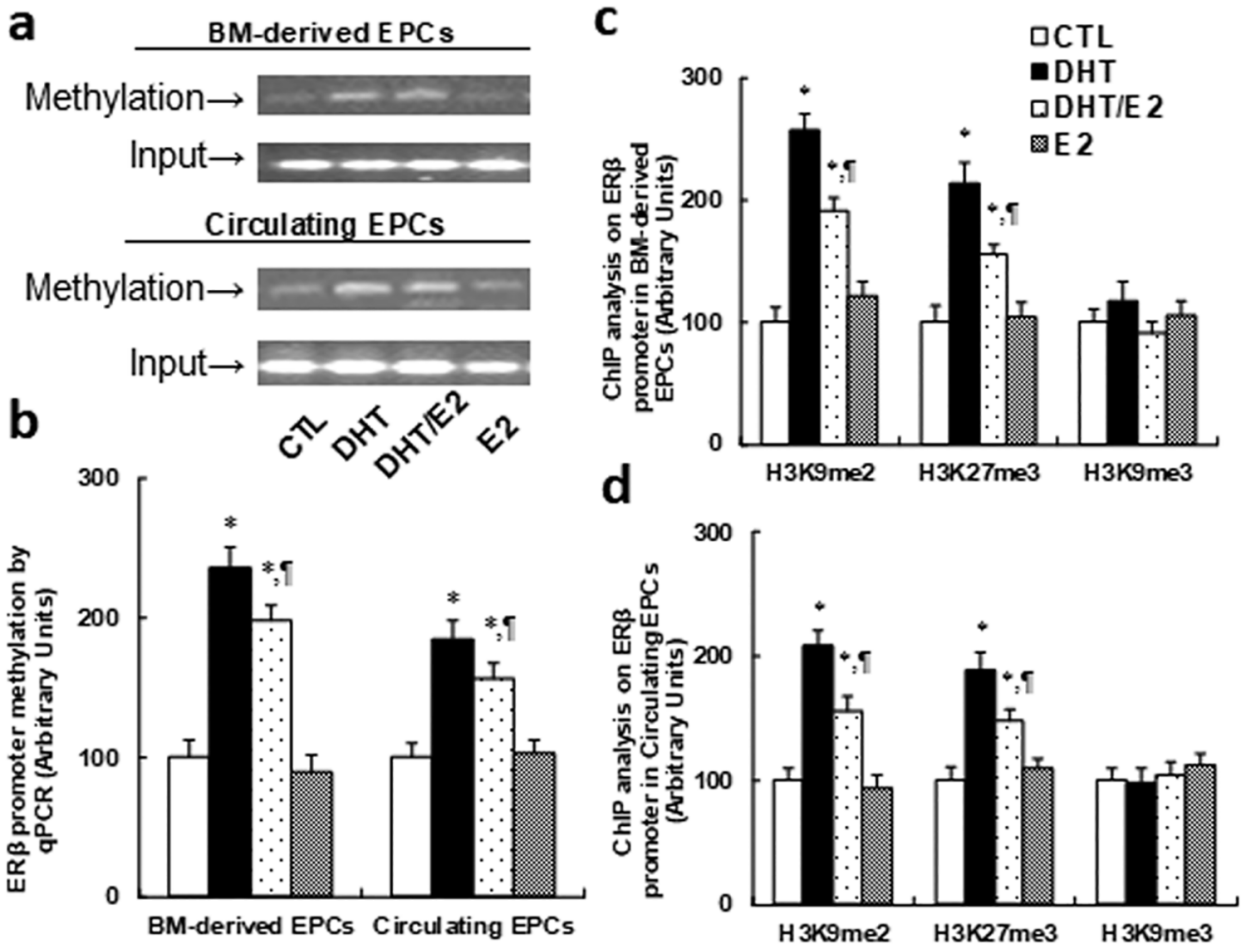
Perinatal testosterone exposure induces ERβ suppression in EPCs through increased methylation on ERβ promoter, while E2 has no effect. The EPCs were isolated from 2-month old male offspring for in vitro cell culture analysis. (a) The representative bands for ERβ methylation in EPCs from male offspring. (b) DNA methylation on ERβ by real-time PCR based methylation specific PCR (MSP) analysis in EPCs, n = 4. (c) ChIP analysis on ERβ promoter in BM-derived EPCs, n = 5. (d) ChIP analysis on ERβ promoter in Circulating EPCs, n = 5. *, *P*<0.05, vs CTL group; ¶, *P*<0.05, vs DHT group. Results are expressed as mean ± SEM.

We measured DNA methylation on the ERβ promoter using methylation specific PCR (MSP) analysis. In Fig 2a and 2b, both DHT and DHT/E2 treatment significantly increased DNA methylation on the ERβ promoter in both bone marrow-derived and circulating EPCs from young male offspring (2 months old), while E2 treatment had no effect. We then measured the epigenetic changes on the ERβ promoter using ChIP techniques. The results showed significant increased H3K9 di-methylation (H3K9me2) and H3K27 tri-methylation (H3K27me3) on the ERβ promoter from both bone marrow-derived EPCs (see Fig 2c) and circulating EPCs (see Fig 2d) with DHT or DHT/E2 treatment in young offspring, while E2 had no effect, indicating that perinatal testosterone exposure-induced ERβ suppression in EPCs is due to increased DNA methylation on the ERβ promoter.

### Perinatal testosterone exposure does not induce ERβ suppression in mouse endothelial cells (MECs) and subsequent vascular dysfunction in young offspring (2 months old)

Since perinatal testosterone exposure induces ERβ suppression in EPCs, we wanted to know whether this affects ERβ expression in MECs and the subsequent vascular function in young offspring (2 months old). We first measured the gene expression of ERβ and its target genes for mRNA (see Panel A in S2 Fig) and proteins level (see Panel B and C in S2 Fig) in MECs, and the results showed no difference in different hormone treatments. We also measured ROS formation (see Panel D in S2 Fig), mitochondrial DNA copies (see Panel E in S2 Fig), intracellular ATP level (see Panel F in S2 Fig), in vitro palmitate acid oxidation (see Panel G in S2 Fig) and in vitro fatty acid uptake (see Panel H in S2 Fig) in MECs, and there was no difference either. We then measured vessel tension (see Panel I in S2 Fig) and blood pressure (see Panel J in S2 Fig) in in vivo mice, and the results showed no difference. This indicates that perinatal testosterone exposure does not induce ERβ suppression in MECs and the subsequent vascular dysfunction in young offspring even though it suppresses ERβ in EPCs.

### Perinatal testosterone exposure induces ERβ suppression and its target genes from both circulating EPCs and MECs in old offspring (20 months old)

**Fig 3:**
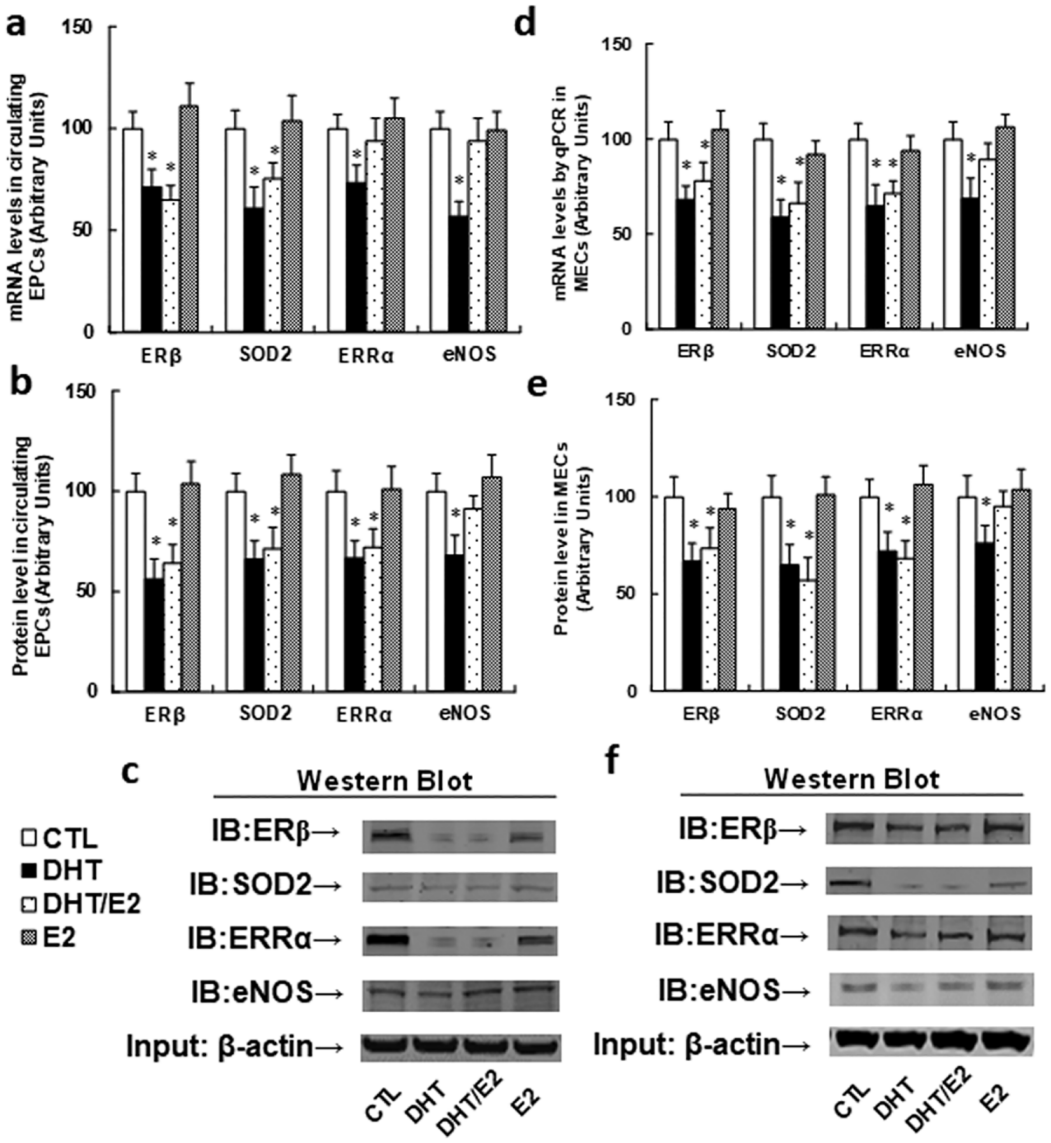
Perinatal testosterone exposure induces ERβ suppression and its target genes from both circulating EPCs and mouse endothelial cells (MECs) in old male offspring (20 months old). (a-c). The EPCs were isolated from treated male offspring for further analysis. (a) mRNA level by qPCR, n = 4. (b) Protein level by western blotting, n = 4. (c) The representative bands for (b). (d) The MECs were isolated from the aorta using Laser Capture Microdissection (LCM) techniques to measure mRNA level by qPCR, n = 5. (e,f) The MECs were isolated from the heart and cultured in vitro for protein analysis using western blotting. (e) Protein level by Western blotting, n = 4. (f) Representative bands for (e). *, *P*<0.05, vs CTL group. Results are expressed as mean ± SEM.

Since we already found that perinatal testosterone exposure induces ERβ suppression in EPCs in young offspring, we wanted to know whether this effect remains in old offspring (24 months old). We measured the ERβ expression for mRNA (see Fig 3a) and protein levels (see Fig 3b and 3c) in circulating EPCs from old offspring, and the results showed that both DHT and DHT/E2 treatment significantly suppressed ERβ expression and its target genes, while E2 treatment showed no effect, indicating that perinatal testosterone exposure-induced ERβ suppression in EPCs lasts for a long time due to DNA methylation on the ERβ promoter. We then measured ERβ expression and its target genes in MECs in old offspring (20 months old), and the results showed that both mRNA (see Fig 3d) and protein levels (see Fig 3e and 3f) were significantly decreased in both DHT and DHT/E2 treatment. This is surprisingly different from MECs in young offspring, which showed no difference on ERβ expression in different treatments, suggesting that perinatal testosterone exposure-induced ERβ suppression in EPCs in young offspring lasts for a long time until old age, and those EPCs with ERβ suppression may mobilize to the vascular wall and differentiate into MECs, which then contributes to the vascular dysfunction.

### Perinatal testosterone exposure induces ROS generation and DNA damage, and dysfunction of mitochondria and fatty acid metabolism in both circulating EPCs and MECs in old offspring (20 months old)

**Fig 4:**
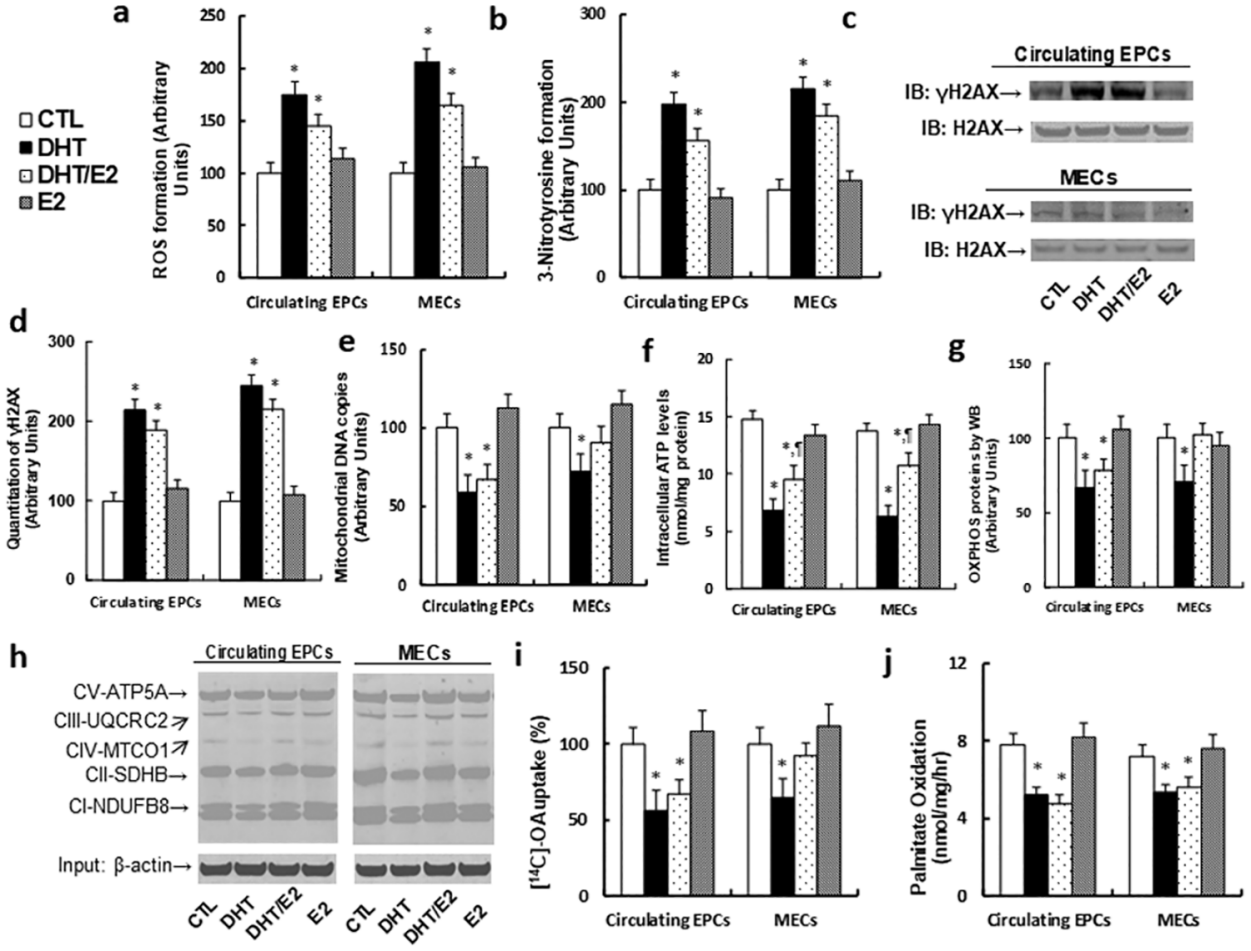
Perinatal testosterone exposure induces ROS generation and DNA damage, and dysfunction of mitochondria and fatty acid metabolism in both circulating EPCs and MECs in old male offspring (20 months old). The MECs from treated mice were isolated from the hearts during 20 months of age for in vitro culture analysis. (a) ROS formation, n = 6. (b) Quantitation of 3-nitrotyrosine (3-NT) formation, n = 4. (c) Representative γH2AX western blotting band. (d) Quantitation of γH2AX formation for (c), n = 5. (e) Mitochondrial DNA copies, n = 4. (f) Intracellular ATP levels, n = 5. (g) Representative western blotting band for OXPHOS proteins. (h) Quantitation of OXPHOS proteins for (g), n = 5. (i) In vitro ^14^C-OA fatty acid uptake, n = 4. (j) The in vitro palmitate oxidation rate, n = 4. *, *P*<0.05, vs CTL group; ¶, *P*<0.05, vs DHT group. Results are expressed as mean ± SEM.

We evaluated the molecular consequences of perinatal testosterone exposure-induced ERβ suppression in both circulating EPCs and MECs in old offspring. In Fig 4, we showed that perinatal testosterone exposure (both DHT and DHT/E2 treatment) significantly increased ROS formation (see Fig 4a), 3-nitrotyrosine formation (see Fig 4b) and DNA damage with γH2AX formation (see Fig 4c and 4d); it also suppressed mitochondrial function, including decreased mitochondrial DNA copies (see Fig 4e), intracellular ATP level (see Fig 4f) and OXPHOS proteins (see Fig 4g and 4h). Furthermore, it suppresses fatty acid metabolism, including decreased in vitro fatty acid uptake (see Fig 4i) and fatty acid oxidation (see Fig 4j). These results indicate that perinatal testosterone exposure-induced ERβ suppression in both circulating EPCs and MECs in old offspring (20 months old) may contribute to vascular dysfunction through ERβ-mediated molecular consequences.

### Perinatal testosterone exposure potentiates vascular dysfunction in old offspring (20 months old), while E2 has no effect

**Fig 5:**
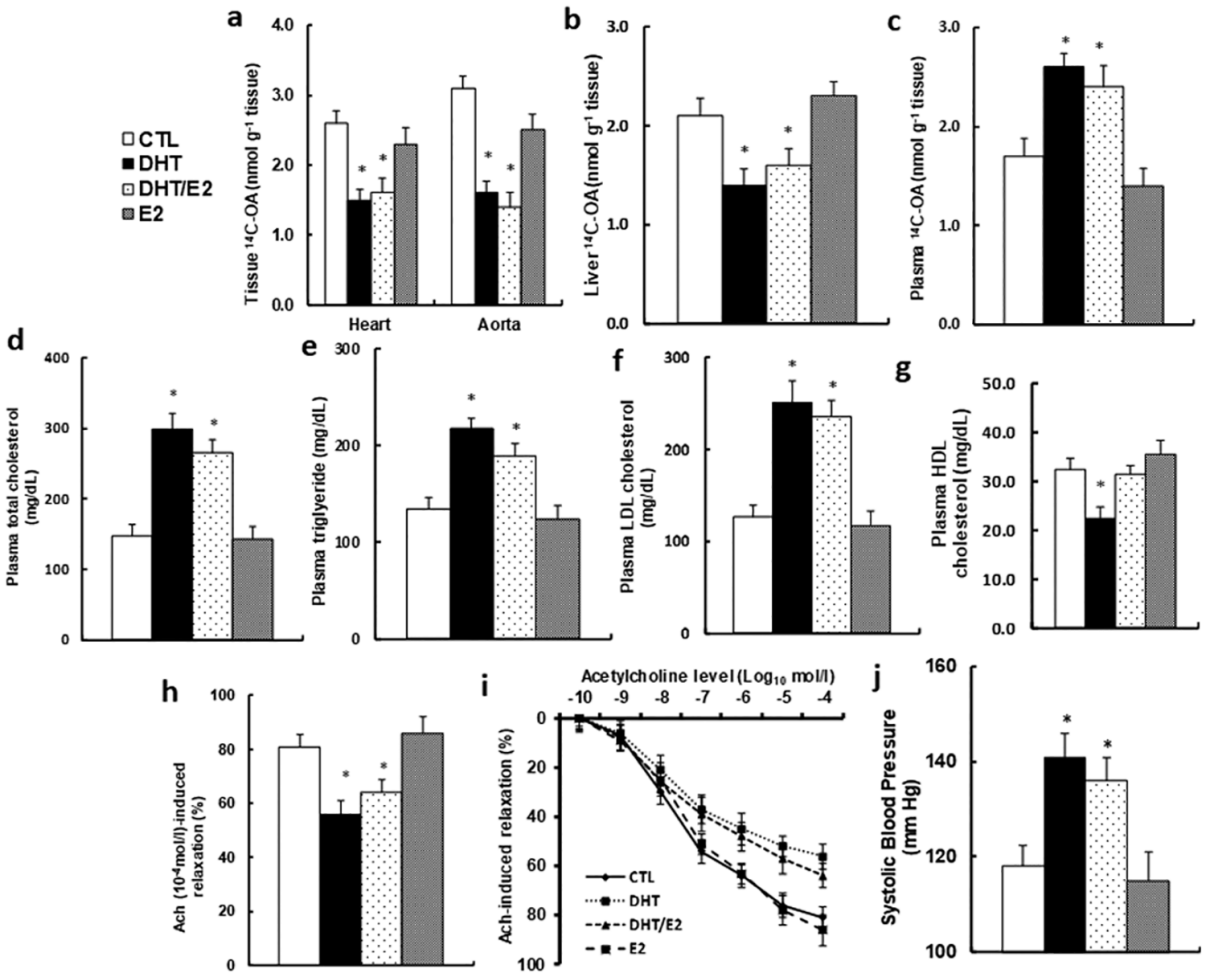
Perinatal testosterone exposure potentiates vascular dysfunction in old male offspring (20 months old). (a-c) The treated male offspring were given a bolus dose of 2mCi of ^14^C-OA through oral gavage, and the blood and tissues, including the heart, aorta and liver, were dissected for analysis of total radioactivity. (a) The in vivo ^14^C-OA uptake from the heart and aorta in 2h, n = 8. (b) The in vivo ^14^C-OA uptake from liver in 2h, n = 7. (c) The in vivo ^14^C-OA uptake in plasma in 1h, n = 6. (d-g) The plasma was collected from treated male offspring for analysis of total cholesterol, n = 10 (d); triglyceride, n = 10 (e); LDL cholesterol, n = 12 (f); and HDL cholesterol, n = 11 (g). (h,i) The aortas were dissected from treated mice for vessel tension analysis. The rings were pre-constricted with phenylephrine, and the acetylcholine (Ach, 10^−10^–10^−4^ mol/l) was injected at the plateau of the phenylephrine-induced contraction. (h) The 10^−4^ mol/l Ach-induced aorta ring relaxation, n = 9–12; (i) The Ach-induced aorta ring relaxation curves. (j) The treated mice were used to measure the mean of systolic blood pressure, n = 11. *, *P*<0.05, vs CTL group. Results are expressed as mean ± SEM.

We evaluated the effect of perinatal testosterone exposure on vascular function in old offspring (20 months old). The in vivo fatty acid metabolism was evaluated using ^14^C-OA uptake, and our results showed that the E2 treatment had little effect, while DHT and DHT/E2 treatment significantly decreased fatty acid uptake in both the heart and aorta (see Fig 5a) and liver (see Fig 5b) with increased plasma fatty acid level (see Fig 5c). This indicates that perinatal testosterone exposure can suppress the in vivo fatty acid metabolism with increased circulating lipids. We then measured the plasma lipids from those old offspring, and the results showed that perinatal testosterone exposure (DHT and DHT/E2 group) significantly increased plasma lipid levels, including total cholesterol (see Fig 5d), triglyceride (see Fig 5e) and LDL cholesterol (see Fig 5f), but with slightly decreased HDL cholesterol levels (see Fig 5g), while E2 treatment showed little effect. We then measured the effect of perinatal testosterone exposure on the changes of vessel tension. In Fig 5h and 5i, acetylcholine-induced relaxation was significantly decreased in DHT and DHT/E2 treatment, and E2 treatment showed no effect. We also measured the changes of blood pressure in Fig 5j. Blood pressure was increased significantly in DHT and DHT/E2 treatment, while E2 treatment had no effect compared to CTL group. Our results indicate that perinatal testosterone exposure-induced ERβ suppression in EPCs may potentiate vascular dysfunction.

### Manipulation of ERβ expression in EPCs through Tie2-driven lentivirus infection on bone marrow-derived MNCs

The Tie2-driven lentivirus for either empty (EMP) control, overexpression of either ERβ or SIRT1-C152(D), or ERβ knockdown (shERβ) was generated to infect MNCs that were isolated from donor mice. In Panel A and B in S3 Fig, the lentivirus pLVX-AcGFP1-C1 was used as a parallel infection of MNCs, and the results showed almost 100% GFP expression with 1.0×10^6^ cfu lentivirus infection, suggesting that our lentivirus infection was sufficient. The lentivirus infected MNCs were isolated for EPCs, and the positive EPCs were identified as Dil-Ac-LDL/Lectin double staining (see Panel C in S3 Fig). The EPCs were cultured in vitro with the presence of 5μg/ml puromycin, the continuous resistance to puromycin reflected a sufficient lentivirus infection. We then measured the gene expression from lentivirus-infected EPCs and non-EPCs that were isolated from MNCs. In Panel D in S3 Fig, the EPCs showed significant suppressed ERβ expression compared to the CTL/EMP group in both T/EMP and CTL/shERβ treatment, and both DHT/↑ERβ and DHT/↑SIRT1-C152(D) treatment significantly increased ERβ expression, while non-EPCs showed only minor changes in ERβ expression. In Panel E in S3 Fig, non-EPCs showed no difference in SIRT1 expression, while in EPCs, only DHT/↑SIRT1-C152(D) treatment significantly increased SIRT1 expression. This indicates that manipulation of ERβ expression in EPCs through Tie2-driven lentivirus infection to MNCs was successful, it was specific in EPCs but not in non-EPCs.

### Bone marrow transplanted EPCs with manipulated ERβ expression mobilize to the vascular wall and differentiate into MECs in old offspring (20 months old)

We measured the molecular consequences in both circulating EPCs and MECs after bone marrow transplantation (BMT) of EPCs with Tie2-driven lentivirus-mediated ERβ expression. We first measured the gene expression in both circulating EPCs and isolated MECs after BMT. We found that ERβ expression was significantly suppressed in DHT/EMP and CTL/shERβ group compared to CTL/EMP group, while it was significantly increased in DHT/↑ERβ and DHT/↑SIRT1-C152(D) group (see Panel A in S4 Fig). On the other hand, SIRT1 expression was only increased in DHT/↑SIRT1-C152(D) group (see Panel B in S4 Fig). Furthermore, the expression level from MECs showed a pattern similar to circulating EPCs. The results indicate that bone marrow transplanted EPCs with lentivirus-driven ERβ expression may be released to the circulation system during old age and mobilize to the vascular wall, differentiate into MECs, and then affect the gene expression of MECs. We then measured the gene expression in MECs and SMCs (smooth muscle cells) that were isolated from aorta using Laser Capture Microdissection (LCM) techniques. In Panel C in S4 Fig, the ERβ expression has no difference in SMCs, indicating that those cells were not affected by perinatal testosterone exposure and subsequent BMT, while in MECs, the ERβ expression was significantly decreased in DHT/EMP and CTL/shERβ treatment compared to CTL/EMP group, and the ERβ expression was significantly restored in DHT/↑ERβ and DHT/↑SIRT1-C152(D) group compared to the DHT/EMP group, indicating that manipulation of ERβ expression by Tie2-driven lentivirus was successful in EPCs and it was mobilized to the vascular wall and differentiated into MECs. In Panel D in S4 Fig, the SIRT1 expression (including SIRT1 wild type and SIRT1 single mutant SIRT1-C152(D)) was significantly increased in MECs from DHT/↑SIRT1-C152(D) treatment, and in Panel E in S4 Fig, the expression of SIRT1-C152(D) was only detected in MECs from DHT/↑SIRT1-C152(D) treatment, but not detected in SMCs. These results indicate that bone marrow transplanted EPCs with ERβ expression only mobilize to the vascular wall and differentiate into MECs, and there is no leaking effect to SMCs.

### Bone marrow transplantation with ERβ overexpression in EPCs restores perinatal testosterone exposure-induced vascular dysfunction, while ERβ knockdown in EPCs worsens the problem

**Fig 6:**
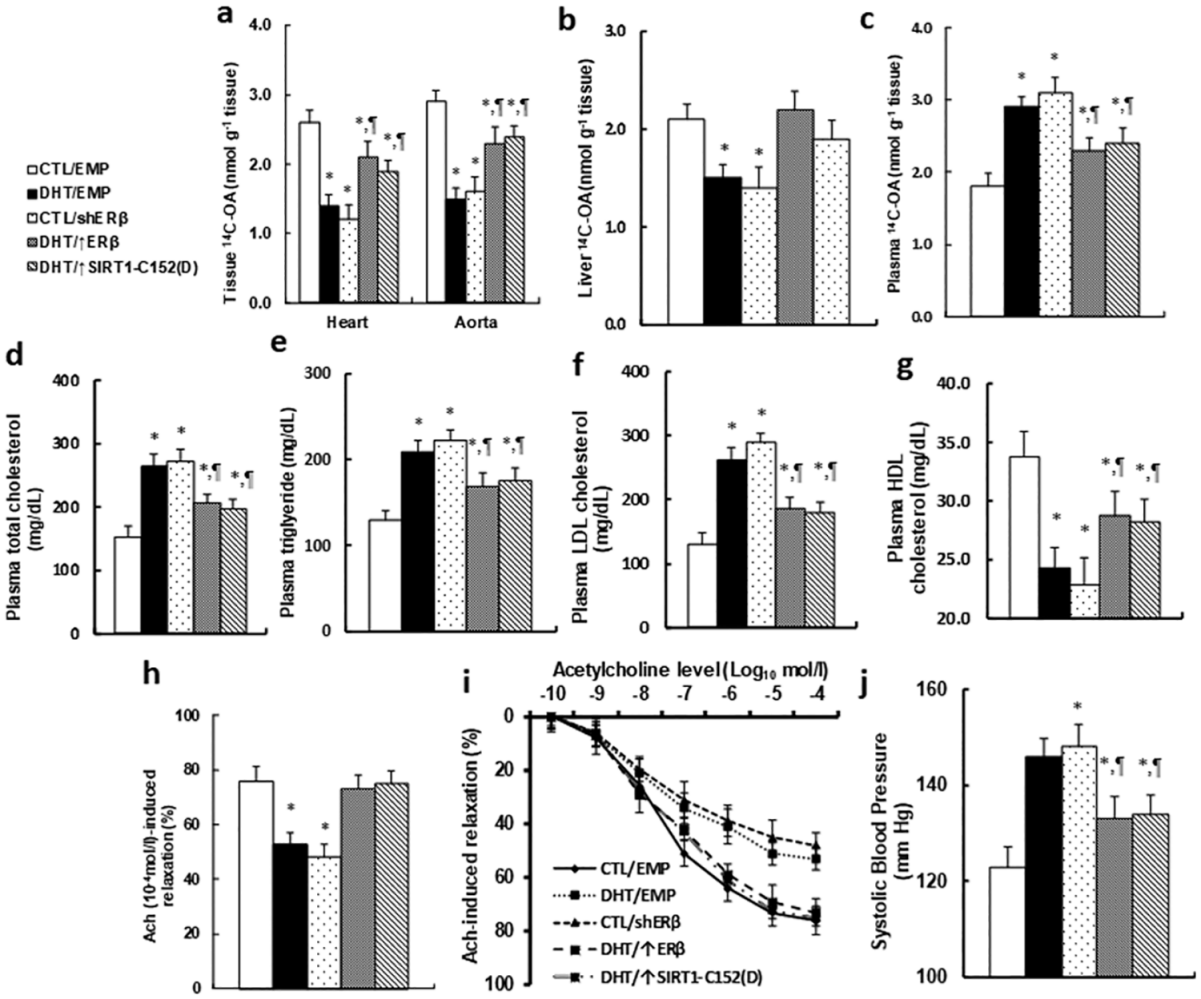
Bone marrow transplantation with ERβ overexpression in EPCs restores perinatal testosterone exposure-induced vascular dysfunction in male old offspring, while ERβ knockdown in EPCs worsens the problem. (a-c) The treated mice were given a bolus dose of 2mCi of ^14^C-OA through oral gavage, and the blood and tissues, including the heart, aorta and liver, were dissected for analysis of total radioactivity. (a) The in vivo ^14^C-OA uptake from the heart and aorta in 2h, n = 6. (b) The in vivo ^14^C-OA uptake from liver in 2h, n = 6. (c) The in vivo ^14^C-OA uptake in plasma in 1h, n = 7. (c) (d-g) The plasma was collected from treated mice for analysis of total cholesterol, n = 9 (d); triglyceride, n = 8 (e); LDL cholesterol, n = 11 (f); and HDL cholesterol, n = 12 (g). (h,i) The aortas were dissected from treated mice for vessel tension analysis. The rings were pre-constricted with phenylephrine, and the acetylcholine (Ach, 10^−10^–10^−4^ mol/l) was injected at the plateau of the phenylephrine-induced contraction. (h) The 10^−4^ mol/l Ach-induced aorta ring relaxation, n = 8–11; (i) The Ach-induced aorta ring relaxation curves. (j) The treated mice were used to measure the mean of systolic blood pressure, n = 10. *, *P*<0.05, vs CTL group; ¶, *P*<0.05, vs DHT group. Results are expressed as mean ± SEM.

The Tie2-driven lentivirus was employed to specifically manipulate ERβ expression in EPCs during bone marrow transplantation (BMT), and the effect of BMT on vascular dysfunction was evaluated. We first evaluated the mobilization characteristics of circulating EPCs from the old male offspring with BMT procedure. The results showed no difference in circulating EPCs numbers (see Panel A in S5 Fig), EPCs colony forming Unit (CFU) (see Panel B in S5 Fig) and EPCs migration (see Panel C in S5 Fig), indicating that manipulation of ERβ expression in bone marrow transplanted EPCs by Tie2-driven lentivirus does not significantly affect mobilization characteristics of circulating EPCs in old offspring (20 months old). We then measured the in vivo fatty acid metabolism using ^14^C-OA uptake. Our results showed that fatty acid uptake was significantly decreased in both the heart and aorta (see Fig 6a) and liver (see Fig 6b) with increased plasma fatty acid level (see Fig 6c) in DHT/EMP and CTL/shERβ group, while DHT/↑ERβ and DHT/↑SIRT1-C152(D) treatment significantly restored this effect. We then measured the plasma lipids from old offspring, and the results showed that DHT/EMP and CTL/shERβ treatment significantly increased plasma lipid levels, including total cholesterol (see Fig 6d), triglyceride (see Fig 6e) and LDL cholesterol (see Fig 6f), but with decreased HDL cholesterol levels (see Fig 6g), and DHT/↑ERβ and DHT/↑SIRT1-C152(D) treatment significantly minimized this effect. We then measured the effect of ERβ expression on the changes of vessel tension. In Fig 6h and 6i, acetylcholine-induced relaxation was significantly decreased in DHT/EMP and CTL/shERβ treatment, while DHT/↑ERβ and DHT/↑SIRT1-C152(D) treatment restored this effect. We also measured the changes of blood pressure in Fig 6j. Blood pressure was increased significantly in DHT/EMP and CTL/shERβ treatment, while DHT/↑ERβ and DHT/↑SIRT1-C152(D) treatment significantly but did not completely restore this effect, indicating that some other factors may also play a role during perinatal testosterone exposure-induced vascular dysfunction. We also briefly repeated the bone marrow transplantation experiments we did on males in female old offspring. In S6 Fig, the DHT/EMP and CTL/shERβ treatment significantly decreased vessel tension (see Panel A in S6 Fig), and increased the systolic blood pressure (see Panel B in S6 Fig), while DHT/↑ERβ and DHT/↑SIRT1-C152(D) treatment partly restored this effect. The results showed similar effects as in male offspring. This indicates that perinatal testosterone exposure-induced ERβ suppression and vascular dysfunction in old offspring has no significant sex difference.

## Discussion

Excessive perinatal exposure to androgens may happen in adult females in polycystic ovary syndrome (PCOS) phenotype with the elevation of androgens during pregnancy [6]. Androgen plays a causative role in the development of vascular dysfunction [2, 35], while the effect of estrogen seems to be the opposite, and plays a protective role in vascular function [11, 12]. In general, the effect of excessive androgens on vascular dysfunction in females remains controversial [36, 37], this can be explained that testosterone (T) can be converted into estradiol (E2) during pregnancy, thus the effects of excess androgen may be partly contributed to by estrogen. Our preliminary experiments showed that perinatal exposure of either DHT alone or combined DHT/E2 treatment, suppressed ERβ expression in EPCs from offspring but not mothers, while E2 exposure alone had no effect. Given the fact that ERβ plays a protective role in vascular function [11–14], we hypothesize that perinatal testosterone exposure may predispose offspring to vascular dysfunction through ERβ suppression in EPCs. In this study, we used 7-week sex hormone exposure including 1-week hormone pellet implantation recovery, 3-week mating and pregnancy, and 3-week lactation in female mice during their pregnancy to mimic the perinatal exposure situation. Also, the dihydrotestosterone (DHT) instead of testosterone (T) was used as androgen treatment to avoid potential effects resulting from aromatization of T to E2 [6].

In this study, we are interested in measuring the potential effect of perinatal testosterone exposure on both male and female offspring, while the male offspring have been selected as the major experimental animal since they do not have too much interference from the endogenous estrogen. On the other hand, we have also briefly repeated the similar experiments in female offspring as shown in results section in S6 Fig, and the results indicate that perinatal testosterone exposure-induced ERβ suppression and vascular dysfunction in old offspring has no significant sex difference. This can be explained that ERβ on vascular wall regulates the basal expression of ERβ target genes, including SOD2 [11] and ERRα [12], which is relatively independent from the activation of estrogen.

### Perinatal testosterone exposure induces DNA methylation and ERβ suppression

Our results showed that perinatal testosterone exposure-induced ERβ suppression in EPCs from young offspring was due to altered methylation on the ERβ promoter. These kinds of epigenetic changes can be heritable in daughter cells, and this is a good explanation for why the same ERβ suppression was observed in EPCs during their old age from those offspring. This is also consistent with the previous finding that epigenetic changes with DNA methylation in ERβ contribute to vascular dysfunction [38]. We also showed that ERβ expression in endothelial cells from vascular wall was decreased during old age even though the ERβ expression was not changed during young age in the same offspring. The difference between young and old offspring on the respects of ERβ expression and the subsequent vascular function can be explained due to the contribution of EPCs functions. We suppose that the EPCs with ERβ suppression mobilized to the vascular wall in response to vascular dysfunction during old age, differentiated into MECs, and eventually contributed to ERβ suppression in MECs, subsequently potentiating vascular dysfunction [39].

### EPCs mobilizes to vascular wall and contributes to the vascular dysfunction

We have previously reported that SIRT1 single mutant SIRT1-C152(D) could restore ERβ suppression in vascular dysfunction through modulation of SIRT1 phosphorylation. In this study, in order to mimic the long lasting epigenetic changes on the ERβ promoter with DNA methylation, we designed a Tie2-driven lentivirus carried ERβ expression system. This kind of virus can be heritable in daughter cells, and is specific in EPCs [14]. When the bone marrow-derived MNCs were infected by the related lentivirus with either Tie2-↑ERβ or Tie2-↑SIRT1-C152(D) for bone marrow transplantation, the ERβ was specifically overexpressed in EPCs, and our results showed that overexpression of either ERβ or SIRT1-C125(D) significantly ameliorated perinatal testosterone exposure-induced vascular dysfunction. We also designed a pair of special real-time PCR primers that are specific for SIRT1-C152(D), instead of SIRT1 wild type (SIRT1-WT). Very interestingly, our results showed that SIRT1-C152(D) was only detectable in MECs, but not in SMCs (smooth muscle cells). This is direct evidence that the Tie2-driven lentivirus infected EPCs in BMT were mobilized to the vascular wall and differentiated into MECs during vascular dysfunction, and there is no leaking effect to SMCs. Our results are consistent with the previous finding, which shows that decreased SIRT1 expression potentiates the senescence of EPCs [40].

## Supporting information

S1 Table Details and conditions for the MNCs in treated mice. (DOCX)

S2 Table Sequences of primers for the real time quantitative PCR (qPCR). (DOCX)

S1 Fig Perinatal testosterone exposure does not significantly affect mobilization characteristics of circulating EPCs in young male offspring (2 months old). (DOCX)

S2 Fig Perinatal testosterone exposure does not induce ERβ suppression in MECs and vascular dysfunction in young male offspring (2 months old). (DOCX)

S3 Fig Manipulation of ERβ expression in EPCs through Tie2-driven lentivirus infection on bone marrow-derived MNCs. (DOCX)

S4 Fig Bone marrow transplanted EPCs with manipulated ERβ expression may mobilize to the vascular wall and differentiate into MECs in old male offspring (20 months old). (DOCX)

S5 Fig Manipulation of ERβ expression in bone marrow transplanted EPCs by Tie2-driven lentivirus does not significantly affect mobilization characteristics of circulating EPCs in old male offspring (20 months old). (DOCX)

S6 Fig Bone marrow transplantation with ERβ overexpression in EPCs restores perinatal testosterone exposure-induced vascular dysfunction in old female offspring, while ERβ knockdown in EPCs worsens the problem. (DOCX)
