# Supplementary material for: Perinatal testosterone exposure potentiates vascular dysfunction by ERβ suppression in endothelial progenitor cells
Source: PLoS One. 2017 Aug 15;12(8):e0182945. doi: 10.1371/journal.pone.0182945 (PMC5557363; doi:10.1371/journal.pone.0182945)
Supplement: S1 Table — (DOCX) [file pone.0182945.s001.docx]

**S1 Table. Details and conditions for the MNCs in treated mice**

| Animal Group | CTL/  EMP | DHT/  EMP | CTL/  shERβ | DHT/  ↑ERβ | DHT/↑SIRT1-C152(D) |
| --- | --- | --- | --- | --- | --- |
| Animal number (n) | 50 | 50 | 50 | 50 | 50 |
| Perinatal exposure of hormones | Vehicle | DHT | Vehicle | DHT | DHT |
| Time of hormone treatment (weeks) | 7 | 7 | 7 | 7 | 7 |
| Tie2-driven  lentivirus infection | Tie2-  Empty | Tie2-  Empty | Tie2-  shERβ | Tie2-  ↑ERβ | Tie2-↑SIRT1-C152(D) |
