# Supplementary material for: Perinatal testosterone exposure potentiates vascular dysfunction by ERβ suppression in endothelial progenitor cells
Source: PLoS One. 2017 Aug 15;12(8):e0182945. doi: 10.1371/journal.pone.0182945 (PMC5557363; doi:10.1371/journal.pone.0182945)
Supplement: S2 Table — (DOCX) [file pone.0182945.s002.docx]

**S2 Table. Sequences of primers for the real time quantitative PCR (qPCR)**

| Gene | Species | Forward primer (5'→3') | Reverse primer (5'→3') |
| --- | --- | --- | --- |
| β-actin | Mouse | tcttgggtatggaatcctgtg | atctccttctgcatcctgtca |
| eNOS | Mouse | gcaggctctcacctacttcct | ctgaaccacttccattcttcg |
| ERβ | Mouse | atgtgctatggccaacttctg | caagcttcctcttcagggtct |
| ERRα | Mouse | caggcttctcctcactgtcac | cccctcttcatctaggaccag |
| SIRT1 | Mouse | gtaagcggcttgagggtaatc | aaacttggactctggcatgtg |
| SOD2 | Mouse | ggcctacgtgaacaatctcaa | tcaggtttgtccagaaaatgg |
