# Supplementary material for: Perinatal testosterone exposure potentiates vascular dysfunction by ERβ suppression in endothelial progenitor cells
Source: PLoS One. 2017 Aug 15;12(8):e0182945. doi: 10.1371/journal.pone.0182945 (PMC5557363; doi:10.1371/journal.pone.0182945)
Supplement: S1 Fig — (DOCX) [file pone.0182945.s003.docx]

**S1 Fig**

**S1 Fig. Perinatal testosterone exposure does not significantly affect mobilization characteristics of circulating EPCs in young male offspring (2 months old).** (a) Number of circulating EPCs in BMT mice, n=5. (b) CFU for circulating EPCs in BMT mice, n=5. (c) Migration assay for circulating EPCs in BMT mice, n=5. Results are expressed as mean ± SEM.
