# Supplementary material for: Perinatal testosterone exposure potentiates vascular dysfunction by ERβ suppression in endothelial progenitor cells
Source: PLoS One. 2017 Aug 15;12(8):e0182945. doi: 10.1371/journal.pone.0182945 (PMC5557363; doi:10.1371/journal.pone.0182945)
Supplement: S2 Fig — (DOCX) [file pone.0182945.s004.docx]

**S2 Fig**

**S2 Fig. Perinatal testosterone exposure does not induce ERβ suppression in MECs and vascular dysfunction in young male offspring (2 months old).** (a-h) The MECs were isolated from the hearts with indicated treatments for further analysis, n=4. (a) mRNA level. (b) Protein level. (c) Representative western blotting band for (b). (d) ROS formation. (e) Mitochondrial DNA copies. (f) Intracellular ATP levels. (g) The in vitro palmitate oxidation rate. (h) In vitro ^14^C-OA fatty acid uptake. (i) The 10^-4^ mol/l Ach-induced aorta ring relaxation from treated mice, n=7. (j) The mean of systolic blood pressure, n=8. Results are expressed as mean ± SEM.
