# Supplementary material for: Perinatal testosterone exposure potentiates vascular dysfunction by ERβ suppression in endothelial progenitor cells
Source: PLoS One. 2017 Aug 15;12(8):e0182945. doi: 10.1371/journal.pone.0182945 (PMC5557363; doi:10.1371/journal.pone.0182945)
Supplement: S3 Fig — (DOCX) [file pone.0182945.s005.docx]

**S3 Fig**

**S3 Fig. Manipulation of ERβ expression in EPCs through Tie2-driven lentivirus infection on bone marrow-derived MNCs.** (a,b) Parallel infection of MNCs cells were achieved using 100μl of 1×10^7^ cfu/ml of pLVX-AcGFP1-C1 virus, and the expressed GFP proteins were photographed and the infection efficiency was calculated. (a) GFP expression photographs. (b) Viral infection efficiency using different lentivirus titers (0.5×10^6^ and 1.0×10^6^ cfu respectively). (c-e) The MNCs were infected by Tie2-driven lentivirus, and were used for isolation of EPCs and non-EPCs. (c) The EPCs were visualized and confirmed by Dil-Ac-LDL/Lectin double staining. (d,e) Both EPCs and non-EPCs cells were used for mRNA analysis of ERβ (d) and SIRT1 (e), n=5. *, *P*<0.05, vs CTL/EMP group; ¶, *P*<0.05, vs DHT/EMP group. Results are expressed as mean ± SEM.
