# Supplementary material for: Perinatal testosterone exposure potentiates vascular dysfunction by ERβ suppression in endothelial progenitor cells
Source: PLoS One. 2017 Aug 15;12(8):e0182945. doi: 10.1371/journal.pone.0182945 (PMC5557363; doi:10.1371/journal.pone.0182945)
Supplement: S4 Fig — (DOCX) [file pone.0182945.s006.docx]

**S4 Fig**

**S4 Fig. Bone marrow transplanted EPCs with manipulated ERβ expression mobilize to the vascular wall and differentiate into MECs in old male offspring (20 months old).** (a,b) The bone marrow transplanted mice were sacrificed at 20 months old, the circulating EPCs and MECs were isolated for in vitro analysis of gene expression of ERβ (a) and SIRT1 (b), n=5. (c-e) The MECs or SMCs (smooth muscle cells) were isolated from aorta using Laser Capture Microdissection (LCM) techniques for mRNA analysis. (c) mRNA level for ERβ, n=5. (d) mRNA level for SIRT1, n=5. (e) mRNA level for SIRT1 single mutant SIRT1-C152(D), n=5. *, *P*<0.05, vs CTL/EMP group; ¶, *P*<0.05, vs DHT/EMP group. Results are expressed as mean ± SEM.
