# Supplementary material for: Perinatal testosterone exposure potentiates vascular dysfunction by ERβ suppression in endothelial progenitor cells
Source: PLoS One. 2017 Aug 15;12(8):e0182945. doi: 10.1371/journal.pone.0182945 (PMC5557363; doi:10.1371/journal.pone.0182945)
Supplement: S5 Fig — (DOCX) [file pone.0182945.s007.docx]

**S5 Fig**

**S5 Fig. Manipulation of ERβ expression in bone marrow transplanted EPCs by Tie2-driven lentivirus does not significantly affect mobilization characteristics of circulating EPCs in old male offspring (20 months old).** (a) Number of circulating EPCs in BMT mice, n=5. (b) CFU for circulating EPCs in BMT mice, n=5. (c) Migration assay for circulating EPCs in BMT mice, n=5. Results are expressed as mean ± SEM.
