# Supplementary material for: Perinatal testosterone exposure potentiates vascular dysfunction by ERβ suppression in endothelial progenitor cells
Source: PLoS One. 2017 Aug 15;12(8):e0182945. doi: 10.1371/journal.pone.0182945 (PMC5557363; doi:10.1371/journal.pone.0182945)
Supplement: S6 Fig — (DOCX) [file pone.0182945.s008.docx]

**S6 Fig**

**S6 Fig. Bone marrow transplantation with ERβ overexpression in EPCs restores perinatal testosterone exposure-induced vascular dysfunction in old female offspring, while ERβ knockdown in EPCs worsens the problem.** (a) The aortas were dissected from treated female offspring (20 months old) for vessel tension analysis. The 10^-4^ mol/l Ach-induced aorta ring relaxation, n=7-9; (b) The treated mice were used to measure the mean of systolic blood pressure, n=7-9. *, *P*<0.05, vs CTL group; ¶, *P*<0.05, vs T group. Results are expressed as mean ± SEM.
